# Pro-inflammatory diets promote the formation of hyperuricemia

**DOI:** 10.3389/fendo.2024.1398917

**Published:** 2024-06-21

**Authors:** Xin Liu, Ting-Yu Chen, Teng-Yu Gao, Ke-Qin Shi, Fu-Qiang Yin, Yun-Xiang Yu, Chao Zhang

**Affiliations:** ^1^ Center for Evidence-Based Medicine and Clinical Research, Taihe Hospital, Hubei University of Medicine, Shiyan, China; ^2^ West China Hospital of Stomatology, Sichuan University, Chengdu, China; ^3^ Department of Orthopedics, Taihe Hospital, Hubei University of Medicine, Shiyan, China

**Keywords:** hyperuricemia, dietary inflammatory index, NHANES, drinking, hypertension, diabetes

## Abstract

**Background:**

Hyperuricemia, as a very prevalent chronic metabolic disease with increasing prevalence year by year, poses a significant burden on individual patients as well as on the global health care and disease burden, and there is growing evidence that it is associated with other underlying diseases such as hypertension and cardiovascular disease. The association between hyperuricemia and dietary inflammatory index (DII) scores was investigated in this study.

**Methods:**

This study enrolled 13, 040 adult subjects (aged ≥ 20 years) from the US National Health and Nutrition Survey from 2003 to 2018. The inflammatory potential of the diet was assessed by the DII score, and logistic regression was performed to evaluate the relationship between the DII score and the development of hyperuricemia; subgroup analyses were used to discuss the influence of other factors on the relationship.

**Results:**

Participants in the other quartiles had an increased risk of hyperuricemia compared to those in the lowest quartile of DII scores. Stratification analyses stratified by body mass index (BMI), sex, hypertension, drinking, diabetes, education level and albumin-creatinine-ratio (ACR) revealed that the DII score was also associated with the risk of hyperuricemia (P<0.05). There was an interaction in subgroup analysis stratified by sex, age, and hypertension (P for interaction <0.05). The results showed a linear-like relationship between DII and hyperuricemia, with a relatively low risk of developing hyperuricemia at lower DII scores and an increased risk of developing hyperuricemia as DII scores increased.

**Conclusions:**

This study showed that the risk of hyperuricemia increased at slightly higher DII scores (i.e., with pro-inflammatory diets), but not significantly at lower levels (i.e., with anti-inflammatory diets). The contribution of the DII score to the development of hyperuricemia increased with higher scores. The relationship between inflammatory diets and hyperuricemia requires more research on inflammation, and this study alerts the public that pro-inflammatory diets may increase the risk of developing hyperuricemia.

## 1 Introduction

Hyperuricemia is a highly prevalent chronic metabolic disease caused by excessive production and/or decreased excretion of uric acid (1). Excessive uric acid accumulation not only causes hyperuricemia, but more seriously will produce gout stones in the joints to exacerbate the damage to the body, and there is accumulating evidence indicating that hyperuricemia is related to chronic kidney disease (2, 3), hypertension, insulin resistance of hypertension (4), cardiovascular disease (5–7), etc. Current research has found a u-shaped relationship between uric acid and all-cause mortality and new evidence-based approaches to the clinical management of hyperuricemia remain elusive (8), while the treatment of asymptomatic hyperuricemia requires further research and debate (9). In the worldwide, especially in emerging countries, the incidence of hyperuricemia is increasing due to changes in factors such as national economies, lifestyles, and dietary patterns, which has a significant impact on not only individual but also global healthcare and the burden of disease (10–12). The prevalence of hyperuricemia in developed countries has largely remained high. In the United States (US), for example, the prevalence of hyperuricemia remained stable at around 20% during the decade 2007–2016 (13, 14).

Uric acid was a product of purine metabolism, which was found in many foods and body tissues (15). Previous studies had shown that high purine diet, excessive alcohol consumption, increased purine metabolism, and tumor lysis syndrome (16) can lead to increased uric acid production. Dietary behavior deserved our attention as a very important controllable factor in uric acid production. Different diets, even the same food, had a more complex mechanism for linking hyperuricemia (15). In addition to high-purine foods (such as animal-derived foods), which can directly raise the purine content of the human body and thus raise the uric acid level, many diets can be associated with hyperuricemia by inducing inflammatory response through intricate mechanisms (17), which was classified as pro- or anti-inflammatory diet depending on their characteristics (18). To specifically estimate the inflammatory potential of various diets, the Dietary Inflammatory Index (DII), also known as the energy-adjusted Dietary Inflammatory Index (e-DII), was applied to score foods in this study (19). Initial studies (20) found that blood C-reactive protein (CRP) varied seasonally and that a high or low dietary inflammation index significantly predicted intervals of CRP levels; and there is a large body of research data (20–22) suggesting that dietary factors play an important role in the regulation of chronic inflammation. In light of this, the original Dietary Inflammatory Index was developed to quantify inflammatory factors in an individual’s diet and to categorize the role of dietary inflammatory factors along a continuum from anti-inflammatory to pro-inflammatory. The DII score was based on the pro- and anti-inflammatory properties of an individual’s overall dietary composition, i.e., macronutrients, micronutrients, and some other dietary components. Previous studies had shown that DII scores were associated with many health conditions in the general population, i.e., high blood pressure, cancer, and even death (23, 24). The complete DII scored the inflammatory potential of 45 foods based on six inflammatory biomarkers (IL-1β, IL-4, IL6, IL-10, TNF-α and CRP): a positive value for the pro-inflammatory diet and a negative value for the anti-inflammatory diet (19). Currently, DII as a tool had been used to assess the inflammatory potential of diet in relation to colorectal cancer (25), cardiovascular disease (26), sleep quality (27) and osteoporosis (28).

Few research had been done on the association between DII and hyperuricemia, more studies are needed to build a more complete and comprehensive knowledge system and implement it in clinical practice. Therefore, the present study was based on the National Health and Nutrition Examination Survey (NHANES) (29), where DII scores were calculated from the foods consumed by participants in the database to demonstrate the inflammatory potential of an individual’s dietary intake, logistic regression modeling was performed to explore the association between dietary inflammation index and the risk of hyperuricemia, and subgroup analyses were performed to demonstrate the degree of influence of the different risk factors, thus providing guidance for clinical practice.

## 2 Materials and methods

### 2.1 Participant selection

NHANES conducted by the Centers for Disease Control’s National Center for Health Statistics is a cross-sectional survey designed to assess the health and nutrition status of adults and children in the US. The survey uses a complex, multistage probabilistic design to provide a nationally representative sample of the non-institutionalized US civilian population, which is unique in that it combines interviews and physical examinations with a variety of laboratory data measurements (http://www.cdc.gov/nchs/nhanes/about_nhanes.htm) (30). A total of 80,312 participants in eight cycles from 2003 to 2018 were involved in this study. All participants’ data were obtained according to the following procedures: after obtaining demographic data and questionnaire data through telephone or home interviews, participants underwent physical examinations and speech indicators at the laboratory or the mobile examination center.

**Figure 1:**
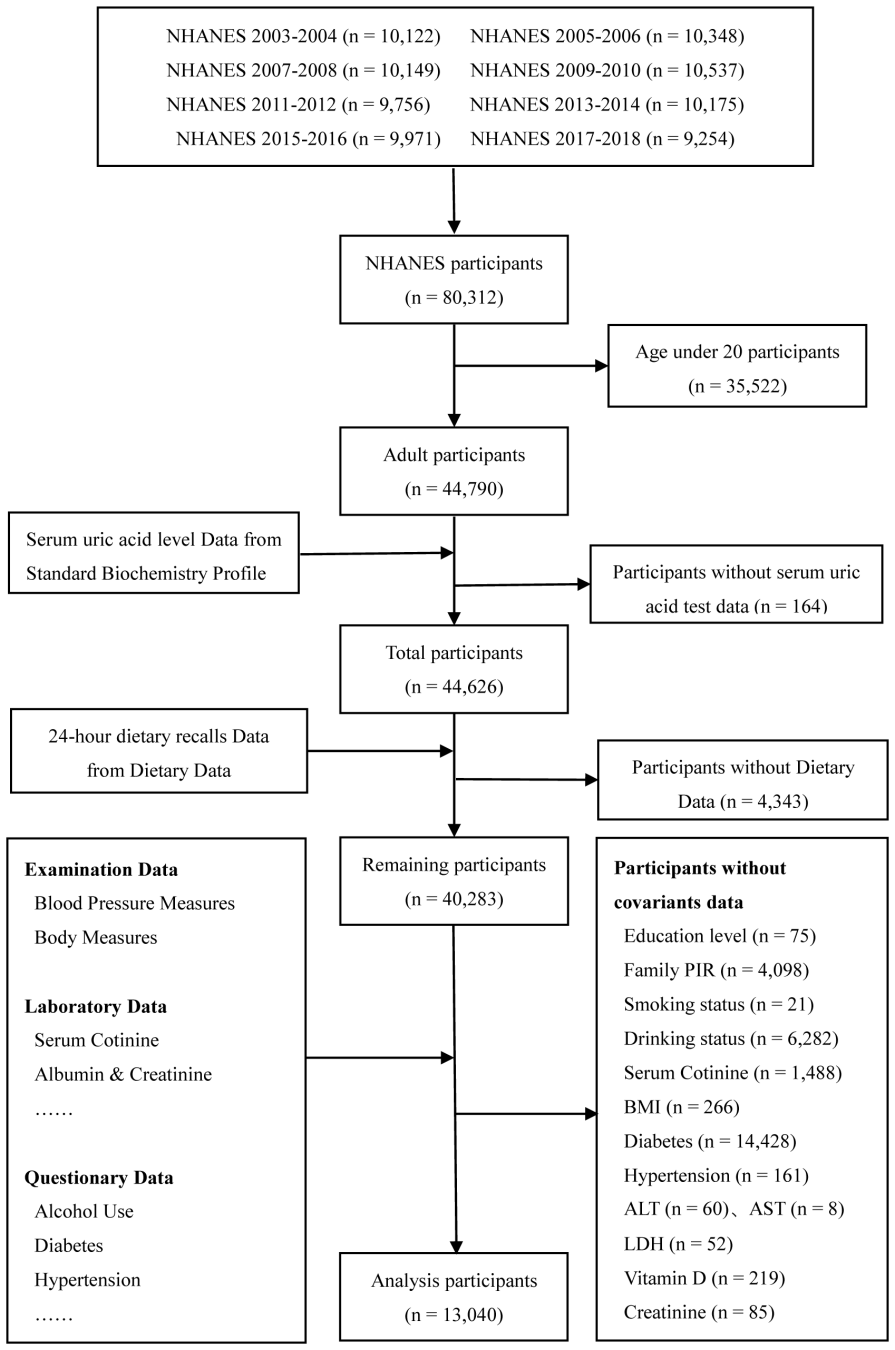
Flow chart for study participant’s selection.

Based on age screening criteria, 35,522 samples that were under 20 years old were not included in this investigation. Since the NHANES database inevitably produces missing and omitted data for some individuals during data collection, the study screened the sample for this inclusion based on strict nerfing criteria, only individuals with complete data were included while those with missing required variables were excluded and the results are as follows: a total of 164 samples without uric acid test data were excluded. Of the 44,626 participants, those without dietary data and those without covariate data (education level, ratio of family income to poverty (family RIP), Body Mass Index (BMI), smoking, serum cotinine, drinking, diabetes, hypertension, alanine transaminase (ALT), lactic dehydrogenase (LDH), vitamin D, and albumin-creatinine-ratio (ACR)) were excluded. Finally, 13,040 adult participants were included in the study, and **Figure 1** showed a flowchart of participant selection.

### 2.2 Assessment of covariates

In the establishment of the model, researcher determined the following covariates based on the variables obtained from the NHANES database, the review of previous research literature, and the preliminary screening by using univariate analysis, taking into account that the test level should be appropriately adjusted when determining covariates and the selection of majors should be combined to avoid missing important risk factors. These covariates, included age, race, sex, BMI, PIR, smoking, drinking, cotinine, blood urea nitrogen (BUN), aspartate aminotransferase (AST), gamma-glutamyl transpeptidase (GGT), ALT, LDH, diabetes, hypertension, and ACR, were evaluated.

### 2.3 Evaluation criterion and inclusion and exclusion criteria

#### 2.3.1 Hyperuricemia

Typically, hyperuricemia was defined as a serum urate level above 6.8 mg/dL (405 µmol/L), as this was the point at which urate solubility was measured in the laboratory using automated enzymatic methods (31). This study, however, did not continue this method of testing uric acid levels. Serum specimens were collected from the participants in the mobile examination center and stored at −20°C until analysis. Then, skilled technicians used the timed endpoint method based on Beckman Coulter UniCel^®^ DxC 800 to analyze the SUA level in the Collaborative Laboratory Services, Ottumwa, Iowa. Because of the role of estrogenic compounds, the definition of hyperuricemia should be determined separately for males and females therefore, in this study, hyperuricemia was defined when serum uric acid concentrations above 7 mg/dl in male adults and above 5.7 mg/dL in female adult (14).

#### 2.3.2 BMI

BMI was defined as weight divided by height squared (kg/m^2^). The included population was classified into three groups according to BMI: normal weight (BMI<25 kg/m^2^), overweight (25≤ BMI<30 kg/m^2^), and obesity (BMI ≥30 kg/m^2^). This criteria is applicable to the diagnosis of overweight, obesity and central obesity in adults (18 years old and above), and can be used for epidemiological screening and clinical preliminary diagnosis.

#### 2.3.3 Drinking

Drinking behavior was classified into two categories. Participants who consumed more than 12 drinks per year were defined as drinkers, and those who consumed no more than 12 drinks per year were considered non-drinkers (32, 33).

#### 2.3.4 Hypertension

Participants’ blood pressure was calculated as the mean of three measurements using standard mercury sphygmomanometers after 10 min of rest. Participants were considered to have hypertension if their systolic blood pressure was ≥130 mmHg and/or diastolic blood pressure was ≥80 mmHg (34). Furthermore, a population currently using antihypertensive drugs was included in this study. The ACR was categorized into three categories (A1: <3 mg/mmol, A2: 3–30 mg/mmol, and A3: >30 mg/mmol) according to guidelines published by the American Chronic Kidney Disease Association (35).

#### 2.3.5 Diabetes

Diabetes was a group of metabolic diseases characterized by hyperglycemia (36). Individuals with fasting blood glucose ≥ 7 mmol/L were considered to have diabetes (37).

### 2.4 Dietary inflammatory index

Briefly, dietary intake data for each study participant were first linked to a database that provided reference global daily means and standard deviation intakes for a total of 45 food parameters from 11 communities worldwide. The z-scores were obtained by subtracting the mean value from the database and then dividing this value by the standard deviation of the parameter. These z-scores were converted to percentile scores and centered on 0 by doubling and subtracting 1 (from -1 to +1). Each central proportion was multiplied by the corresponding literature-derived inflammatory effect score for each food parameter. Finally, the overall DII score for each individual was calculated as the sum of the DII scores for each food parameter.

In general, the DII parameter was based on 45 different foods (38). Based on earlier research, DII scores based on fewer than 30 items were still dependable even if the NHANES program only recorded 26 foods (39–42). In this study, the dietary data used to calculate the DII score were evaluated using the average of two 24-h dietary recalls. All NHANES participants were eligible for two 24-hour dietary recall interviews. The first dietary recall interview was collected in the Mobile Examination Center (MEC) and the second interview is collected by telephone 3 to 10 days later. In brief, individual foods/beverages taken during the 24-hour period preceding the interview were gathered through face-to-face interviews (the first 24-h dietary recall interview) and telephone interviews (the second 24-h dietary recall interview). The parameters, including protein, total fat, cholesterol, dietary fiber, carbohydrate, energy, saturated, monounsaturated, and polyunsaturated fatty acids, ω-3 and ω-6 polyunsaturated fatty acids, vitamin A, vitamin B1, vitamin B2, vitamin B6, vitamin B12, vitamin C, vitamin E, folic acid, alcohol, caffeine, iron, magnesium, zinc, selenium and β-carotene, were used to calculate the DII scores in this study. In this study, all subjects were divided into four groups (Q1: ≤-0.58; Q2: -0.58, 0.26; Q3:0.26 to 0.98; Q4: ≥0.98) according to the quartile of DII.

### 2.5 Statistical analysis

For the categorical variables, percentages were employed to describe them, and for the continuous variables, means and standard deviations were employed. T-test (43), Wilcoxon rank-sum test (44), or Chi-square test was conducted to determine statistical significance based on the hyperuricemia and non-hyperuricemia groups.

Odds ratios (OR) with 95% confidence intervals (CI) of DII quartiles and hyperuricemia were estimated using logistic regression, and the values of P for trend were tested across quartiles. Three statistical models (45, 46) were established (Model I was unadjusted, Models II and III were adjusted, and Models II and III were adjusted for different covariates in each group). Multivariable logistic regression models were established to investigate the association between DII and hyperuricemia in specimens with different parameter levels, including sex, BMI, drinking, hypertension, diabetes, education level and ACR. Subgroup analyses, including sex, age, race, smoking status, PIR, diabetes, hypertension, BMI, education level and ACR, were conducted to further explore the association between DII and hyperuricemia. Furthermore, smooth curve fitting (penalty spline method) was performed to explain the association between the DII scores and hyperuricemia. The R 4.2.2 software (R Foundation for Statistical Computing, Vienna, Austria) was used for all statistical analyses, and P<0.05 was considered statistically significant.

## 3 Results

### 3.1 Characteristics of study population

The demographic characteristics of the participants aged 20 years and older were outlined in **Table 1**. Overall, 13,040 participants were categorized into the non-hyperuricemia group (n=10,151) and the hyperuricemia group (n=2,889). For all individuals, the population with hyperuricemia was older, had a larger percentage of non-Hispanic white people, and had higher levels of BMI, ALT, AST, BUN, GGT, LDH, and ACR (P<0.05). It was worth mentioning that the population with hyperuricemia had higher DII scores than those without hyperuricemia (P<0.05).

**Table 1:** Baseline characteristics of participants from NHANES databases 2003–2018.

Characteristics	Without Hyperuricemia (n=10,151)	Hyperuricemia (n=2,889)	P
Age, mean (SE)	46.71 (16.68)	51.65 (17.33)	<0.001
<45	31.77 (7.23)	31.99 (7.24)	
45–65	54.29 (5.92)	55.53 (5.79)	
>65	73.40 (5.22)	73.88 (5.21)	
Gender, (%)			0.775
Female	51.6	51.1	
Male	48.4	48.9	
Race, (%)			<0.001
Hispanic	13.3	9.2	
Non-Hispanic Black	9.6	11.3	
Non-Hispanic White	70.5	72.5	
Other Race	6.6	7.1	
Education level (%)			0.206
College or above	61.7	60.1	
GED or Equivale	23.2	25.2	
Less than high school	15.0	14.7	
PIR, (%)			0.334
<1	13.3	12.0	
1–2	20.6	22.0	
2–4	29.6	30.4	
>4	36.6	35.6	
Drink, (%)			0.421
Yes	72.0	70.8	
No	28.0	29.2	
Smoker, (%)			<0.001
Current	21.2	17.7	
Former	25.0	31.1	
Never	53.8	51.3	
Cotinine, mean (SE)	58.42 (126.26)	54.30 (125.88)	0.328
BMI, mean (SE)	28.00 (6.28)	32.48 (7.50)	<0.001
ALT, mean (SE)	24.48 (21.39)	29.06 (19.89)	<0.001
AST, mean (SE)	24.55 (18.81)	27.45 (15.92)	<0.001
BUN, mean (SE)	12.88 (4.57)	15.29 (6.77)	<0.001
GGT, mean (SE)	25.71 (37.18)	35.94 (54.22)	<0.001
LDH, mean (SE)	128.19 (28.65)	134.38 (30.29)	<0.001
Diabetes, (%)			<0.001
Yes	8.10	12.5	
No	91.9	87.5	
Hypertension, (%)			<0.001
Yes	40.9	65.5	
No	59.1	34.5	
Perfluorooctane sulfonate, mean (SE)	11.38 (14.09)	13.40 (17.72)	0.044
ACR, mean (SE)	23.94 (207.59)	69.35 (400.16)	<0.001
MiBp, mean (SE)	10.04 (14.02)	10.92 (16.54)	0.211
VID3, mean (SE)	67.48 (27.64)	65.26 (28.45)	0.049
DII, mean (SE)	0.05 (1.06)	0.21 (1.07)	<0.001

SE, standard error; ACR, Albumin creatinine ratio; PIR, ratio of family income to poverty; GED, General educational development; BMI, body mass index; ALT, alanine transaminase; AST, aspartate aminotransferase; BUN, blood urea nitrogen; GGT, gamma-glutamyl transpeptidase; LDH, lactic dehydrogenase; MiBp, mono-isobutyl phthalate; VID3, vitamin D3; DII, dietary inflammatory index. P<0.05 indicated statistically significant.

### 3.2 Association between DII and hyperuricemia

As shown in **Table 2**, multivariable logistic regression analyses were used to examine the relationship between the DII and hyperuricemia. In Model I (unadjusted), compared with those in the Q1 quartile, participants in the other three groups (Q2: OR:1.16, 95%CI=1.03–1.31; Q3: OR:1.32, 95%CI=1.17–1.49; Q4: OR:1.58, 95%CI=1.41–1.78; P for trend <0.05) had a higher risk of hyperuricemia. In Model II (adjusted for age, race, sex), compared to those in the first quartile (Q1 group), the Q2 group had no statistical significance (Q2: OR:1.12, 95%CI=0.99–1.27), while the Q3 and Q4 groups had a higher potential for hyperuricemia (Q3: OR:1.25, 95%CI=1.10–1.41; Q4: OR:1.41, 95%CI=1.24–1.59; P for trend <0.05). In Model III (adjusted for Family PIR, BMI, education level, drinker, smoker, cotinine, ALT, AST, BUN, GGT, LDH, diabetes, hypertension, and ACR in addition to Model II), the Q2 group had no statistical significance (Q2: OR:1.05, 95%CI=0.92–1.20), while the Q3 and Q4 groups had a higher risk for hyperuricemia (Q3: OR:1.19, 95%CI=1.04–1.36; Q4: OR: 1.33, 95%CI=1.16–1.52; P for trend <0.05).

**Table 2:** Associations between hyperuricemia and DII.

Models	DII, OR (95%CI)				P for trend
	Q1 (≤-0.58)	Q2 (-0.58 - 0.26)	Q3 (0.26 - 0.98)	Q4 (≥0.98)	
Model I	Reference	1.16 (1.03,1.31)	1.32 (1.17,1.49)	1.58 (1.41,1.78)	<0.001
Model II	Reference	1.12 (0.99,1.27)	1.25 (1.10,1.41)	1.41 (1.24,1.59)	<0.001
Model III	Reference	1.05 (0.92,1.20)	1.19 (1.04,1.36)	1.33 (1.16,1.52)	<0.001

CI, confidence interval; DII, dietary inflammatory index; OR, odds ratio. Model I: Unadjusted; Model II: Adjusted for age, race, and sex; Model III: Adjusted for family PIR, BMI, alcohol consumption, smoking, education level, cotinine, ALT, AST, BUN, GGT, LDH, diabetes, hypertension, ACR, and Model II. P<0.05 indicated statistically significant.

### 3.3 Subgroup analysis

Subgroup analyses conducted according to the categories stratified by sex, age, race, smoking, PIR, diabetes, hypertension, BMI, education level and ACR were shown in **Table 3**. The positive associations between DII and hyperuricemia were found in female (OR: 1.15, 95%CI=1.07–1.24, P<0.05), male (OR:1.07, 95%CI=1.01–1.14, P<0.05), participants aged 45 to 60 years (OR: 1.13, 95%CI=1.04–1.22, P<0.05), non-Hispanic white population (OR:1.09, 95%CI=1.02–1.17, P<0.05), non-Hispanic black population (OR: 1.17, 95%CI=1.06–1.30, P<0.05), participants with former smoking (OR: 1.12, 95%CI=1.03–1.21, P<0.05), participants with never smoking (OR:1.23, 95%CI=1.05–1.20, P<0.05), participants with 1≤PIR<2 (OR:1.10, 95%CI=1.01–1.20, P<0.05), participants with PIR>4 (OR:1.11, 95%CI=1.01–1.21, P<0.05), participants without diabetes (OR: 1.06, 95%CI=1.05–1.15, P<0.05), participants with hypertension (OR:1.10, 95%CI=1.04–1.17, P<0.05), participants with 25≤BMI<30 (OR:1.11, 95%CI=1.03–1.21, P<0.05), participants with lower (less than high school: OR=1.13, 95%CI=1.06–1.21, P<0.05) or higher (college or above: OR=1.12, 95%CI=1.01–1.24, P<0.05) education level and participants with moderate ACR level (OR=1.09, 95%CI=1.04–1.15, P<0.05). Moreover, in the subgroup analysis stratified by sex, age, and hypertension, the association between the DII and hyperuricemia (P for interaction <0.05) was more pronounced.

**Table 3:** Subgroup analysis of confounding factors.

Confounding factors	Uric Acid (mean (mim, max)), mg/dL		β1 (95% CI)	P for β	P for interaction
	Hyperuricemia	Without hyperuricemia			
Sex					0.009
Female	6.76 (5.8, 12.3)	4.36 (1.1, 5.7)	1.151 (1.073, 1.236)	<0.001	
Male	7.95 (7.1, 17.6)	5.61 (0.8, 7.0)	1.072 (1.008, 1.140)	0.027	
Age					<0.001
<45	4.85 (1.2, 7.0)	7.28 (5.8, 17.6)	1.067 (0.985, 1.156)	0.114	
45–65	5.04 (0.8, 7.0)	7.27 (5.8, 11.7)	1.125 (1.044, 1.213)	0.002	
>65	5.13 (1.1, 7.0)	7.41 (5.8, 13.0)	1.070 (0.978, 1.170)	0.140
Race/Ethnicity					0.136
Mexican American	4.89 (1.6, 7.0)	7.29 (5.8, 17.6)	1.072 (0.969, 1.188)	0.179	
Non-Hispanic white	5.01 (1.1, 7.0)	7.30 (5.8, 12.3)	1.090 (1.020, 1.165)	0.011	
Non-Hispanic black	4.97 (1.4, 7.0)	7.37 (5.8, 11.7)	1.174 (1.059, 1.304)	0.002	
Other Races	5.00 (0.8, 7.0)	7.29 (5.8, 10.9)	0.985 (0.849, 1.145)	0.848	
Smoking					0.099
Current	5.04 (0.8, 7.0)	7.32 (5.8, 12.2)	0.993 (0.891, 1.107)	0.902	
Former	5.15 (1.7, 7.0)	7.46 (5.8, 17.6)	1.115 (1.025, 1.214)	0.012	
Never	4.86 (1.1, 7.0)	7.23 (5.8, 13.0)	1.123 (1.054, 1.197)	<0.001	
Poverty income ratio					0.811
<1	4.89 (1.6, 7.0)	7.26 (5.8, 17.6)	1.096 (0.980, 1.227)	0.112	
1–2	4.94 (0.8, 7.0)	7.37 (5.8, 12.3)	1.100 (1.007, 1.203)	0.036	
2–4	5.00 (1.4, 7.0)	7.30 (5.8, 13.0)	1.080 (0.989, 1.180)	0.086	
>4	5.04 (1.1, 7.0)	7.29 (5.8, 10.3)	1.106 (1.014, 1.208)	0.024	
Diabetes					0.121
Diabetes	5.08 (2.0, 7.0)	7.43 (5.8, 12.3)	1.060 (0.936, 1.202)	0.358	
Without Diabetes	4.96 (0.8, 7.0)	7.29 (5.8, 17.6)	1.098 (1.045, 1.154)	<0.001	
Hypertension					0.003
Hypertension	5.17 (1.1, 7.0)	7.40 (5.8, 17.6)	1.101 (1.039, 1.167)	0.001	
Without hypertension	4.81 (0.8, 7.0)	7.12 (5.8, 13.0)	1.059 (0.982, 1.144)	0.139	
BMI					0.985
<25	4.69 (1.1, 7.0)	7.20 (5.8, 11.9)	1.108 (0.988, 1.244)	0.081	
25–30	5.06 (0.8, 7.0)	7.38 (5.8, 12.3)	1.112 (1.025, 1.207)	0.011	
≥30	5.16 (1.8, 7.0)	7.31 (5.8, 17.6)	1.068 (1.003, 1.138)	0.040	
Education level					0.849
College or above	4.94 (0.8, 7.0)	7.28 (5.8, 17.6)	1.119 (1.012, 1.238)	0.029	
GED or Equivale	5.02 (1.4, 7.0)	7.39 (5.8, 11.7)	0.987 (0.897, 1.086)	0.789	
Less than high school	5.00 (1.9, 7.0)	7.31 (5.8, 13.0)	1.131 (1.063, 1.205)	<0.001	
ACR category					0.149
A1	5.44 (1.2, 7.0)	7.50 (5.8, 11.2)	1.077 (0.900, 1.289)	0.420	
A2	4.93 (0.8, 7.0)	7.22 (5.8, 13.0)	1.091 (1.035, 1.150)	0.001	
A3	5.01 (1.1, 7.0)	7.59 (5.8, 17.6)	1.105 (0.980, 1.246)	0.103	

1, Effect size; ACR, Albumin creatinine ratio; BMI, Body mass index; CI, Confidence interval; DII, Dietary inflammatory index; P<0.05 indicated statistically significant; P for interaction <0.05 indicated statistically significant.

### 3.4 Stratification analysis stratified by gender

**Figure 2:**
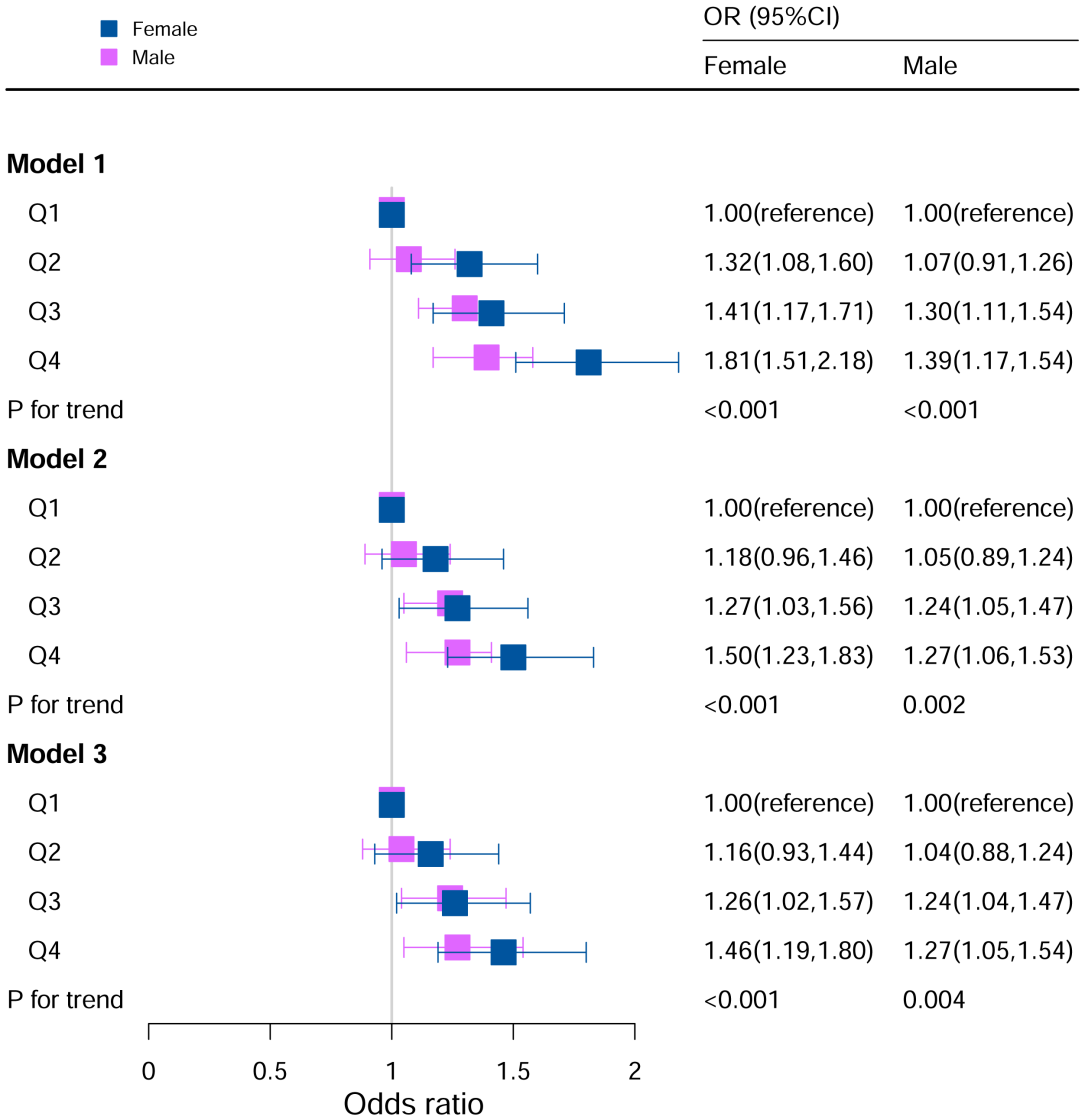
Associations between hyperuricemia and DII stratified by gender. CI, confidence interval; DII, Dietary Inflammatory Index, OR odds ratio. Model I: Unadjusted; Model II: Adjusted for age, race, BMI; Model III: Adjusted for family PIR, education level, BMI, drinking, smoking, cotinine, ALT, AST, BUN, GGT, LDH, diabetes, hypertension, and ACR in addition to Model II.

The stratification analysis in **Figure 2** demonstrated the association between the DII and hyperuricemia stratified by sex. For females, in Model I (unadjusted), participants in the other quartiles were at a higher risk of hyperuricemia (Q2: OR:1.32, 95%CI=1.08–1.60; Q3: OR:1.41, 95%CI=1.17–1.71; Q4: OR:1.81, 95%CI=1.51–2.18; P for trend <0.05). In Model II (adjusted for age, race, BMI), the Q3 and Q4 quartile groups had a higher risk of hyperuricemia than Q1 (Q3: OR:1.27, 95%CI=1.03–1.56; Q4: OR:1.50, 95%CI=1.23–1.83; P for trend <0.05). In Model III (adjusted for family PIR, BMI, education level, drinking, smoking, cotinine, ALT, AST, BUN, GGT, LDH, diabetes, hypertension, and ACR in addition to Model II), the Q3 and Q4 quartiles groups had a higher risk of hyperuricemia than Q1 (Q3: OR: 1.28, 95%CI=1.03–1.59; Q4: OR: 1.49, 95%CI=1.21–1.84; P for trend <0.05).

For the male in Model I, participants in Q3 and Q4 were at a higher risk for hyperuricemia (Q3: OR:1.30, 95%CI=1.11–1.54; Q4: OR:1.39, 95%CI=1.17–1.54; P for trend <0.05). In Model II (adjusted for age, race, BMI), the Q3 and Q4 quartiles had a higher risk of hyperuricemia than Q1 (Q3: OR:1.24, 95%CI=1.05–1.47; Q4: OR:1.27, 95%CI=1.06–1.53; P for trend <0.05). In Model III (adjusted for family PIR, BMI, education level, drinking, smoking, cotinine, ALT, AST, BUN, GGT, LDH, diabetes, hypertension, and ACR in addition to Model II), the Q3 and Q4 quartiles had a higher risk of hyperuricemia (Q3: OR:1.24, 95%CI=1.04–1.48; Q4: OR: 1.29, 95%CI=1.06–1.56; P for trend <0.05).

### 3.5 Stratification analysis stratified by BMI

When BMI was <25, Model I (unadjusted) (Q4: OR:1.49, 95%CI=1.11–2.01) and Model II (adjusted for age, race, sex) (Q4: OR:1.43, 95%CI=1.04–1.95) in the Q4 group were statistically significant.

When BMI was 25 to 30, for Model I (unadjusted), OR of Q3 and Q4 were higher than Q1 (Q3: OR: 1.43, 95%CI=1.15–1.77; Q4: OR: 1.55, 95%CI=1.25–1.93; P for trend <0.05). After adjusting for potential covariates, the OR of Q3 and Q4 were higher than those of Q1.

**Figure 3:**
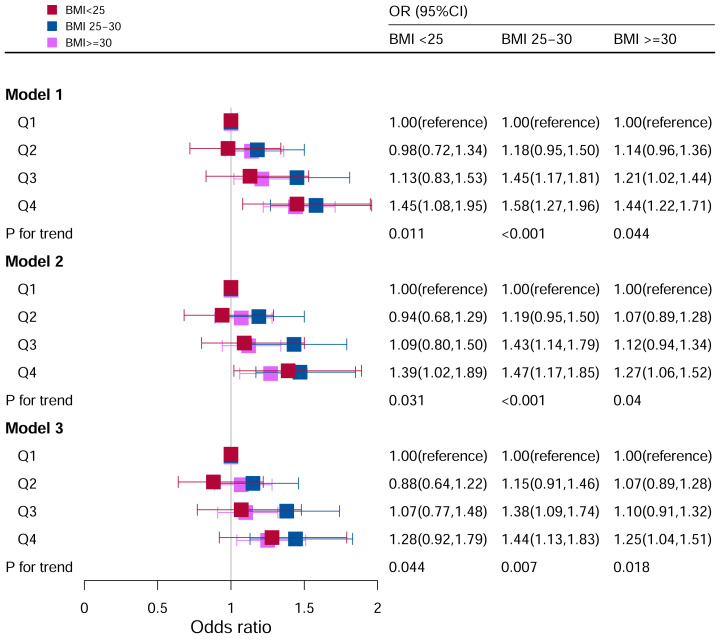
Associations between hyperuricemia and DII stratified by BMI. CI, confidence interval; DII, Dietary Inflammatory Index, OR odds ratio. Model I: Unadjusted; Model II: Adjusted for age, race, and sex; Model III: Adjusted for family PIR, education level, drinking, smoking, cotinine, ALT, AST, BUN, GGT, LDH, diabetes, hypertension, and ACR in addition to Model II.

When BMI ≥30, for Model I (unadjusted), subjects in Q4 had 44% higher odds of OR than those in Q1 (Q4: OR:1.44, 95%CI=1.22–1.71, P for trend <0.05). In Model II (adjusted for age, race, sex), subjects in Q4 had 27% higher odds of OR than those in Q1 (Q4: OR:1.27, 95%CI=1.06–1.52, P for trend <0.05). In Model III (adjusted for family PIR, education level, drinking, smoking, cotinine, ALT, AST, BUN, GGT, LDH, diabetes, hypertension, and ACR in addition to Model II), the population in Q4 had 27% higher odds of OR than those in Q1 (Q4: OR: 1.27, 95%CI=1.05–1.53, P for trend <0.05) (**Figure 3**).

### 3.6 Stratification analysis stratified by hypertension

**Figure 4:**
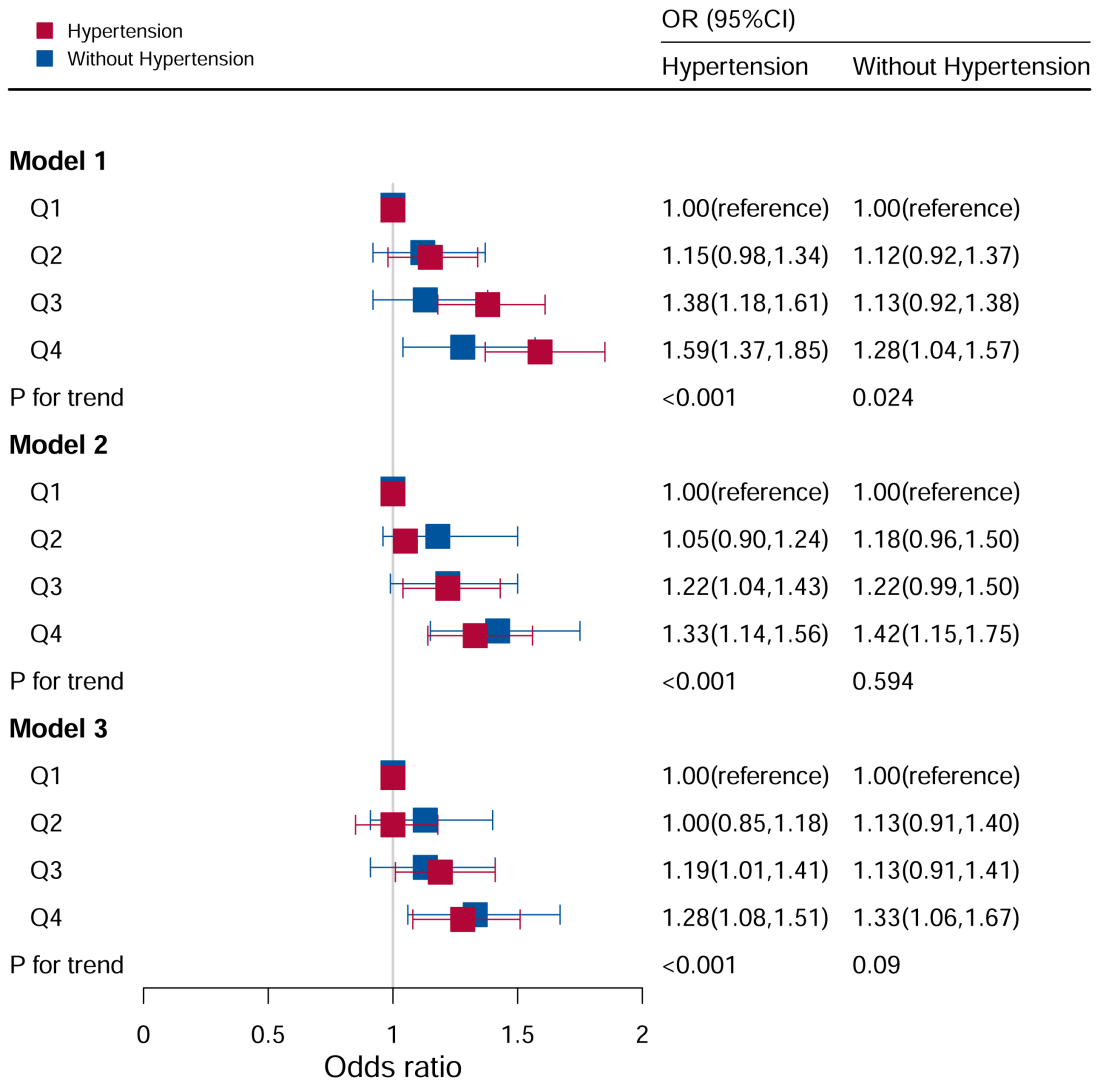
Associations between hyperuricemia and DII stratified by hypertension. CI, confidence interval; DII, Dietary Inflammatory Index, OR odds ratio. Model I: Unadjusted; Model II: Adjusted for age, race, and sex; Model III: Adjusted for family PIR, education level, BMI, drinking, smoking, cotinine, ALT, AST, BUN, GGT, LDH, diabetes, and ACR, in addition to Model II.

In the analysis stratified by hypertension (**Figure 4**), all participants were categorized into hypertensive and non-hypertensive groups. In the hypertensive population, Model I revealed that there was an impressive difference between increased odds of hyperuricemia and higher DII scores (Q3: OR:1.38, 95%CI=1.18–1.61; Q4: OR:1.59, 95%CI=1.37–1.85; P for trend <0.05). After adjusting for age, race, and sex, the Q3 and Q4 quartiles had a higher risk of hyperuricemia (Q3: OR:1.22, 95%CI=1.04–1.43; Q4: OR: 1.33, 95%CI=1.14–1.56). In Model III (adjusted for family PIR, education level, BMI, drinking, smoking, cotinine, ALT, AST, BUN, GGT, LDH, diabetes and ACR in addition to Model II), the Q3 and Q4 quartiles had a higher risk of hyperuricemia (Q3: OR:1.20, 95%CI=1.01–1.42; Q4: OR: 1.28, 95%CI=1.08–1.52).

In the non-hypertensive group, the OR of the Q4 group was statistically significant based on Model I (Q4: OR: 1.28; 95%CI=1.04–1.57). After adjusting for potential confounding factors, the association between hyperuricemia and high DII levels (Q4: OR=1.35, 95%CI=1.07–1.69) remained significant in the non-hypertensive group, while no significant associations were observed with other DII levels.

### 3.7 Stratification analysis stratified by drinking

**Figure 5:**
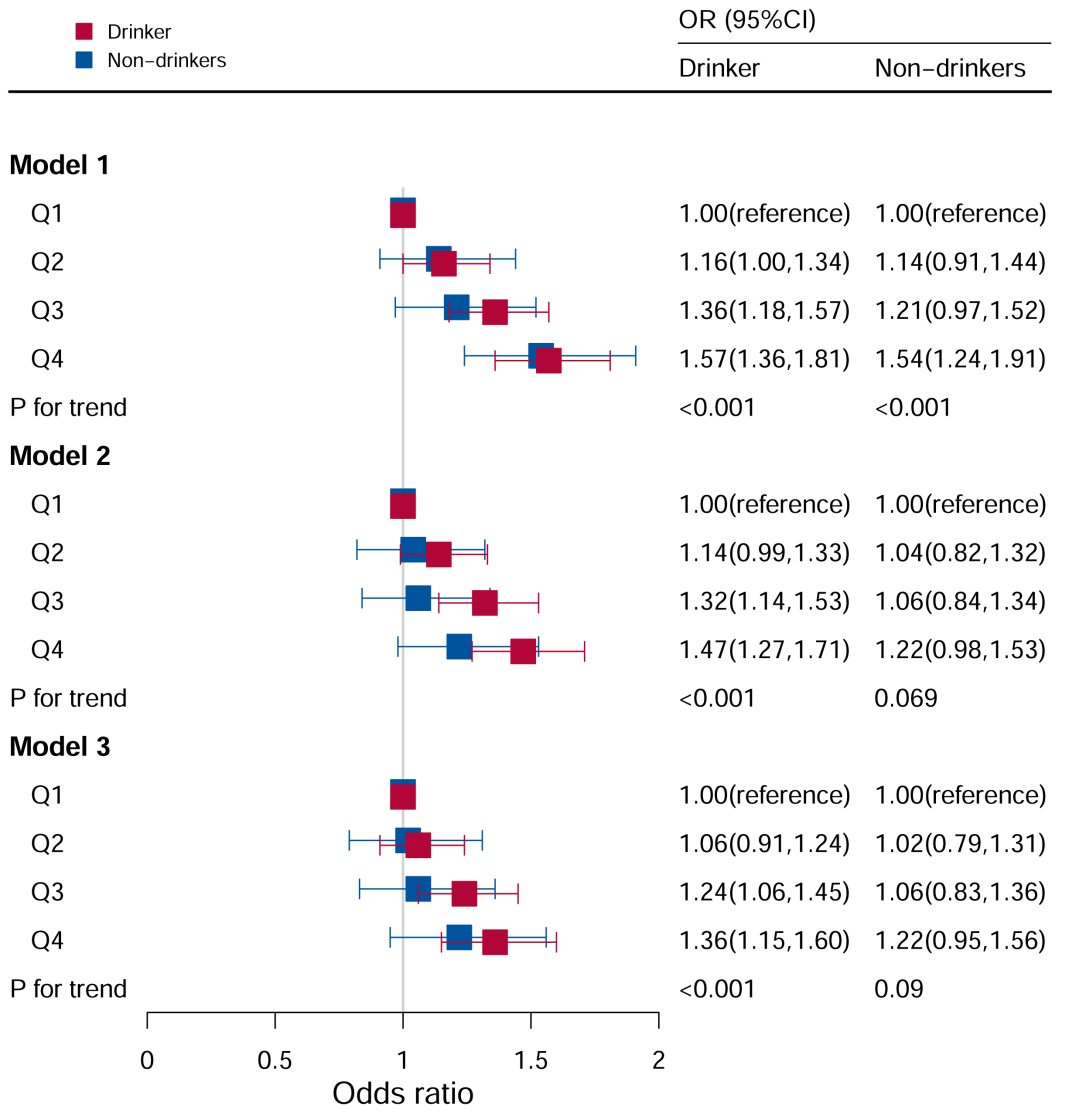
Associations between hyperuricemia and DII stratified by drinking. CI, confidence interval; DII, Dietary Inflammatory Index, OR odds ratio. Model I: Unadjusted; Model II: Adjusted for age, race, and sex; Model III: Adjusted for family PIR, education level, BMI, smoking, cotinine, ALT, AST, BUN, GGT, LDH, diabetes, hypertension, and ACR in addition to Model II.

As shown in **Figure 5**, it observed a significant correlation between DII and hyperuricemia levels in models I, II, and III, and the risk of hyperuricemia increased as DII levels increase. In unadjusted Model I (P for trend <0.05), the Q3 and Q4 quartiles had a higher risk of hyperuricemia (Q3: OR:1.36, 95%CI=1.18–1.57; Q4: OR: 1.57, 95%CI=1.36–1.81). In Model II (P for trend <0.05), the Q3 and Q4 quartiles had a higher risk of hyperuricemia (Q3: OR:1.32, 95%CI=1.14–1.53; Q4: OR: 1.47, 95%CI=1.27–1.71). After adjusting for all covariates, the Q3 and Q4 quartiles had a higher risk of hyperuricemia (Q3: OR:1.24, 95%CI=1.06–1.46; Q4: OR: 1.37, 95%CI=1.16–1.61) in Model III (P for trend <0.05). In contrast, there was no statistical correlation between DII levels and hyperuricemia in participants who did not drink alcohol (P for trend = 0.081).

### 3.8 Stratification analysis stratified by diabetes

**Figure 6:**
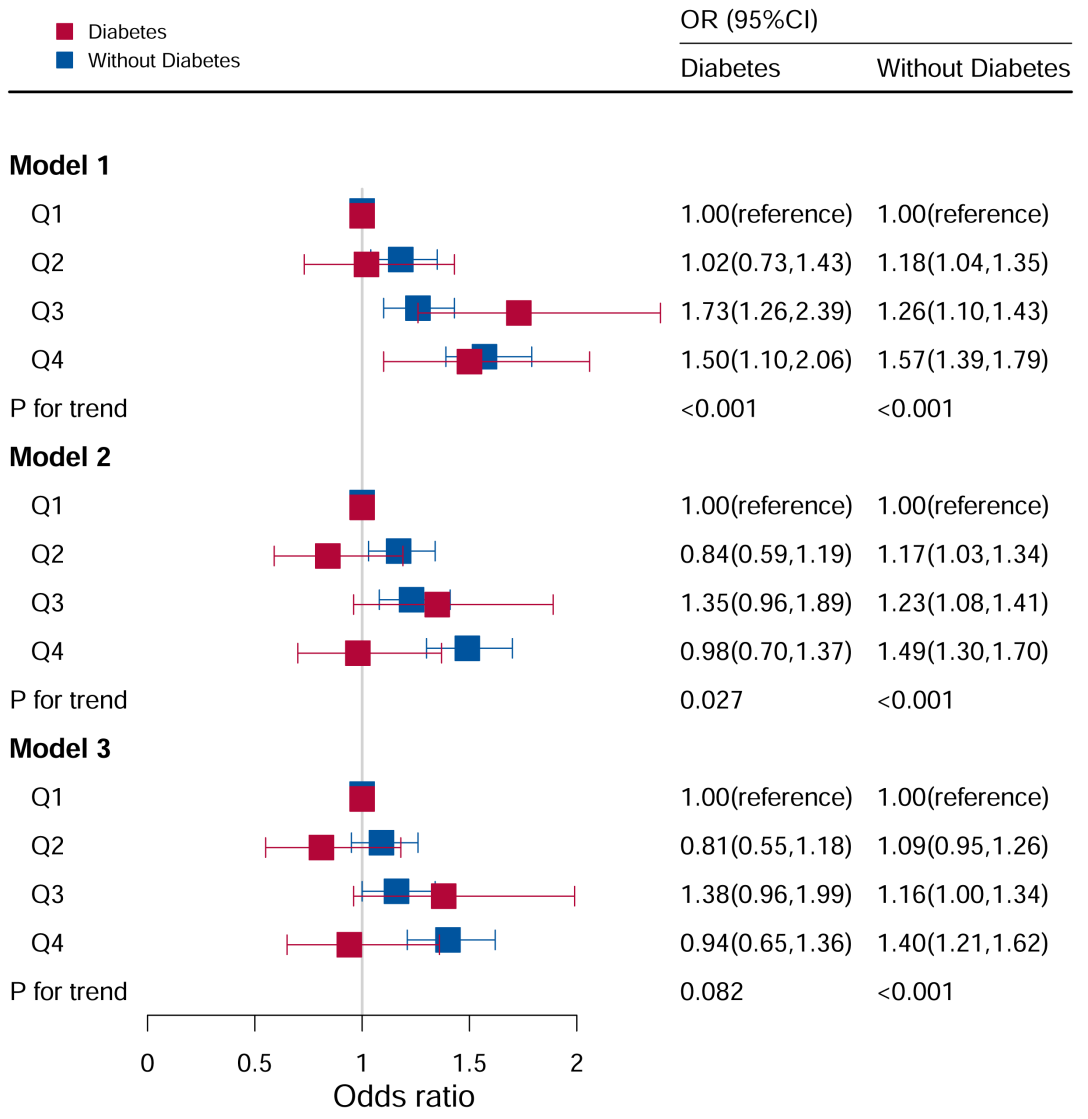
Associations between hyperuricemia and DII stratified by diabetes. CI, confidence interval; DII, Dietary Inflammatory Index, OR odds ratio. Model I: Unadjusted; Model II: Adjusted for age, race, and sex; Model III: Adjusted for family PIR, education level, BMI, drinking, smoking, cotinine, ALT, AST, BUN, GGT, LDH, hypertension, and ACR in addition to Model II.

The association between the DII and hyperuricemia stratified by diabetes according to the quartile of the DII score were shown in **Figure 6**. In the group with diabetes mellitus, after adjusting for all potential risk factors, it wasn’t found that DII levels were associated with hyperuricemia (P for trend =0.506).

In the population without diabetes, the risk of hyperuricemia increased with an increase in DII score quartile. Furthermore, in Model I (P for trend <0.05), the risk of hyperuricemia increased by 57% (OR:1.57, 95%CI=1.39–1.79) in comparing Q4 to Q1. After adjusting for age, race, and sex (P for trend <0.05), the risk of hyperuricemia increased by 49% (OR:1.49, 95%CI=1.30–1.70) when comparing Q4 to Q1. After adjusting for family PIR, education level, BMI, drinking, smoking, cotinine, ALT, AST, BUN, GGT, LDH, hypertension, and ACR in addition to Model II (P for trend <0.05), the risk of hyperuricemia increased by 40% (OR:1.40, 95%CI=1.21–1.62) in Q4 compared to Q1.

### 3.9 Stratification analysis stratified by education level

**Figure 7:**
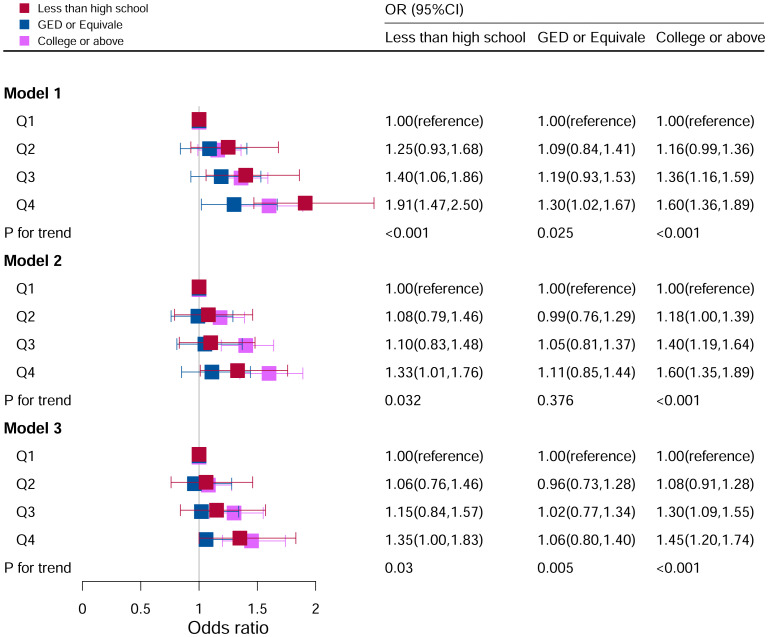
Associations between hyperuricemia and DII stratified by education level. CI, confidence interval; DII, Dietary Inflammatory Index, OR odds ratio. Model I: Unadjusted; Model II: Adjusted for age, race, and sex; Model III: Adjusted for family PIR, BMI, drinking, smoking, cotinine, ALT, AST, BUN, GGT, LDH, hypertension, and ACR in addition to Model II.

**Figure 7** showed the relationship between DII levels and hyperuricemia for various educational groups. It found a statistically significant correlation between DII levels and hyperuricemia only among participants in the university and above education group (P for trend <0.05). After adjusting all covariates, the higher the DII level, the higher the risk of hyperuricemia. The risk of disease in Q4 (OR=1.45, 95%CI=1.20–1.74) group was 45% higher than that in Q1 group. However, there was no significant association between DII score and hyperuricemia in the population with lower education level.

### 3.10 Stratification analysis stratified by albumin-to-creatinine ratio

**Figure 8:**
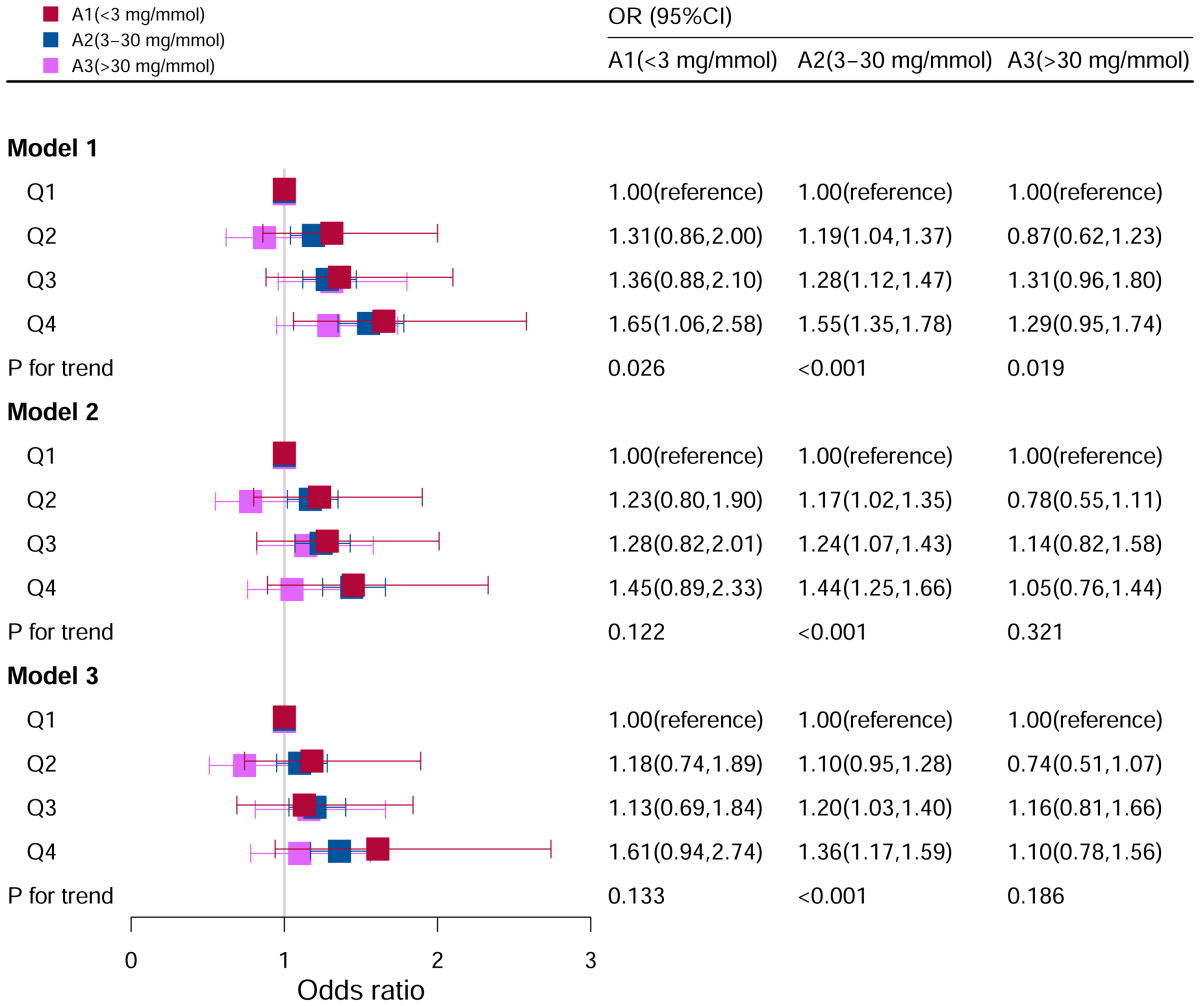
Associations between hyperuricemia and DII stratified by albumin creatinine ratio. CI, confidence interval; DII: Dietary Inflammatory Index, OR odds ratio. Model I: Unadjusted; Model II: Adjusted for age, race, and sex; Model III: Adjusted for family PIR, education level, BMI, drinking, smoking, cotinine, ALT, AST, BUN, GGT, LDH and hypertension in addition to Model II.

**Figure 8** showed the relationship between DII levels and hyperuricemia under different ACR level groupings. No statistical correlations were found after adjusting for all the potential risk factors in subgroups of A1 (<3 mg/mmol) (P=0.133) and A3 (>30 mg/mmol) (P=0.186). Whereas in subgroup of A2 (3–30 mg/mmol), it was found that the risk factor of hyperuricemia was higher with increasing DII levels, and it was a significant statistical correlation (P for trend <0.05). Compared to Q1 level in the model III, the risk of hyperuricemia was increased by 36% at Q4 level (OR=1.36, 95%CI=1.17–1.59).

### 3.11 Curve fitting analysis

**Figure 9:**
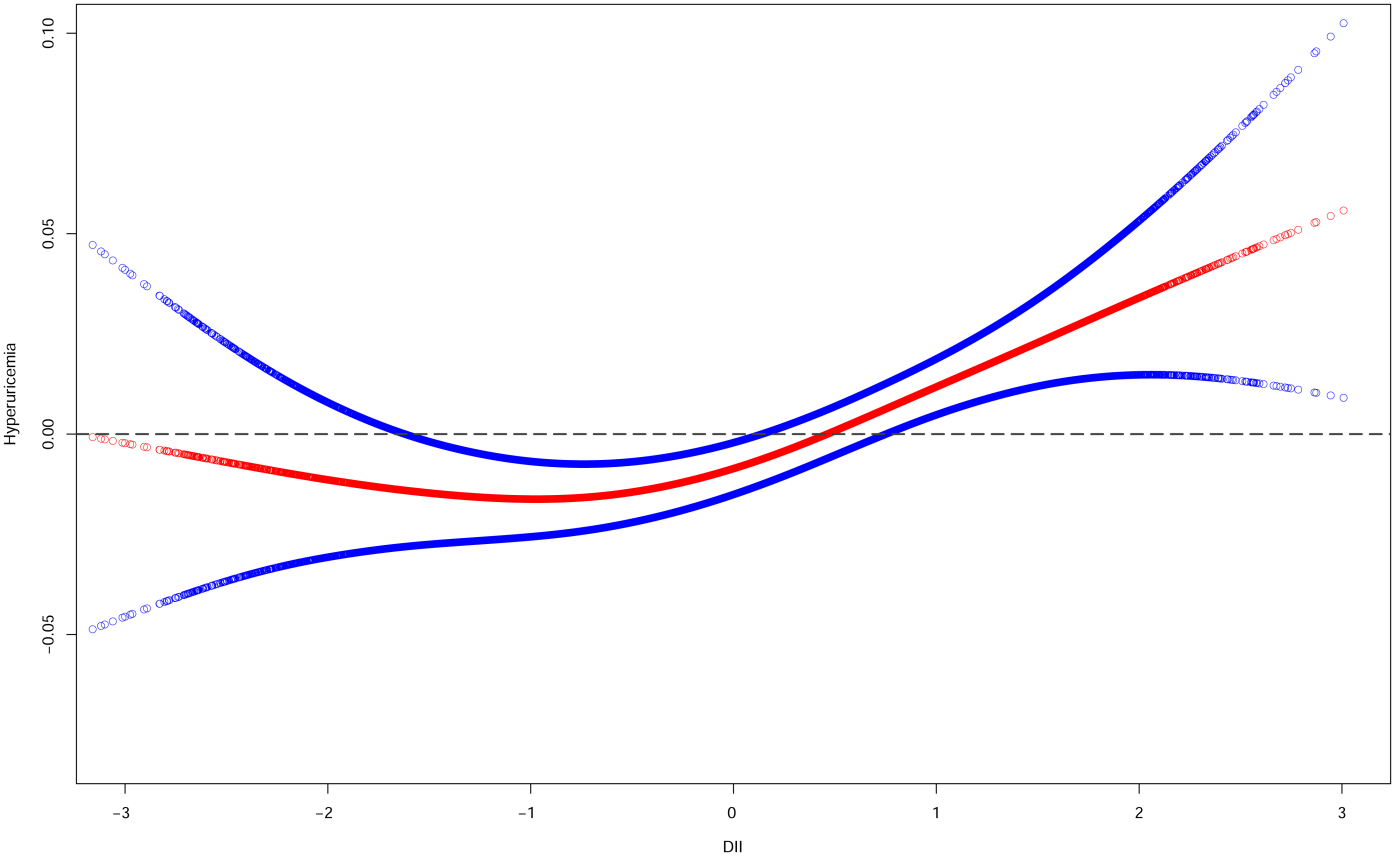
Smooth curve fittings of DII and hyperuricemia. The red curve represents the relationship between DII and hyperuricemia, and the area between the blue dashed lines represents the 95% confidence interval obtained from the fit.

The relationship between DII and hyperuricemia was classically linear by smoothed curve fitting (**Figure 9**). At lower levels of the DII score, there was no significant increase in the risk of hyperuricemia, but as the DII score increased, so did the risk of hyperuricemia, especially at higher levels of the DII score.

## 4 Discussion

This study found that the DII score was associated with hyperuricemia. Further stratification analysis, including sex, hypertension, drinking, BMI, diabetes, education level and ACR. This study indicated that the DII score was strongly associated with hyperuricemia, and the likelihood of hyperuricemia increased with the intake of more pro-inflammatory foods, this conclusion is consistent with the findings of Chen Ye et al. (47) in their investigation of dietary inflammatory index and risk of hyperuricemia in adult Chinese residents, in addition to the study by Hao Wang et al. (48). Especially in females, males, aged 45–65, non-Hispanic white population, non-Hispanic black population, individuals who had smoked in the past and those who had never smoked, individuals with 1≤PIR<2 and PIR>4, individuals without diabetes, individuals with hypertension, individuals with 25≤BMI<30 and BMI>30, individuals with a college education or above and less than high school and participants with moderate ACR.

Diet had long been of interest to researchers as a risk factor that was highly correlated with many diseases and can be controlled by artificial interventions. Current studies suggested that the intake of red meat, seafood, alcohol, or fructose may increase the risk of hyperuricemia, whereas the intake of dairy products or soy foods may reduce the risk of hyperuricemia (49). The association between high protein, coffee diet, and hyperuricemia differed between males and females (49). A cross-sectional analysis showed that higher consumption of soft drinks and fructose was associated with a higher risk of hyperuricemia (50). These discoveries made it worthwhile to conduct more in-depth studies on the relationship between the inflammatory effects associated with diet and the risk of hyperuricemia, providing new perspectives on clinical disease management.

The sex-stratified results of the stratification analysis demonstrated that the prevalence of hyperuricemia was positively correlated with DII in all populations, and it was more obvious in females. Moreover, in subgroup analyses, women were more sensitive to dietary inflammation than men. Nitric oxide (NO) may be a potential influencing factor in the gender-specific differences in uric acid metabolism, with previous studies suggesting that Estrogen can mediate and regulate the expression of nitric oxide synthase (eNOS) through genes, and eNOS is the source of NO produced by endothelial cells (51, 52). Estradiol may affect serum uric acid levels by affecting renal function (53). However, previous studies had shown that only progestin was found to reduce uric acid levels, whereas estradiol intake was not associated with uric acid reduction in females (54, 55). Therefore, the role of sex differences in the occurrence of hyperuricemia needed to be further investigated.

The relationship between BMI and hyperuricemia showed a positive association, indicating that body fat was related to the risk of hyperuricemia and that obesity may serve as a link between diet and hyperuricemia (56). Wang et al. showed that the higher the BMI, the higher the serum uric acid level in a healthy population (57). The association between obesity and serum uric levels may be related to superfluous uric acid productivity and poor renal excretion. This suggested that weight loss and weight loss diets may help prevent hyperuricemia.

As we all know, alcohol can increase uric acid in serum, which was a proven risk factor for hyperuricemia (58–60). Alcohol was associated with hyperuricemia by increasing purine content and/or participating in inflammation. Our study also validated the results of previous studies in the alcoholic vs. non-alcoholic hyperuricemia group. Therefore, it was recommended to reduce or stop all types of alcohol intake to reduce the risk of hyperuricemia or to help patients with hyperuricemia to better treat and slow the progression of hyperuricemia.

In the stratification analysis stratified by hypertension, the DII score was associated with hyperuricemia. A statistically significant correlation between the DII score and hyperuricemia risk was found in participants with hypertension based on the subgroup analysis and interaction test, suggesting an interaction between hypertension and DII. At present, an etiological link between uric acid and hypertension had been established based on epidemiological and clinical data, but the exact mechanism of this association had not been well established. Several cross-sectional, cohort, and interventional studies had reported hyperuricemia as an independent risk factor for hypertension (61–65). The biological basis of uric acid-induced hypertension may affect endothelial cell function and reduce NO (66).

In this study, we observed a positive correlation between a slightly elevated pro-inflammatory diet and the prevalence of hyperuricemia in diabetic patients. Conversely, among the non-diabetic population, an increased level of pro-inflammatory diet was associated with a higher risk of developing hyperuricemia. The causes of diabetes included, but were not limited to, insulin resistance, which referred to the reduced sensitivity of cells to glucose uptake or insulin stimulation when faced with normal or elevated glucose concentrations, and was more common in individuals with type 2 diabetes, obesity, and hypertension (67). If insulin resistance was present, pancreatic β-cells must secrete more insulin, resulting in compensatory hyperinsulinemia. Previous studies had shown that hyperinsulinemia may regulate hypertension by activating renin-angiotensin, further reducing renal blood flow, and increasing the reabsorption of urate and xanthine, leading to hyperuricemia (68). Moreover, there may be a bidirectional causal relationship between hyperuricemia and insulin resistance. A recent study investigating the role of uric acid in glucose metabolism based on a uricase-gene-deficient hyperuricemia mouse model suggested that the relationship between hyperuricemia and diabetes may not be mediated through islet β-cell survival (69). This finding is partially in dispute with the results of this study. Although the study found that a slightly elevated level of a pro-inflammatory diet was also associated with an increased risk of hyperuricemia in diabetic patients, the increased risk of hyperuricemia was more pronounced at higher levels of a pro-inflammatory diet. Since the relationship between diabetes and hyperuricemia and the etiology of both need to be further confirmed, therefore, the correlation underlying the association between hyperuricemia and diabetes required further attention.

Educational background as a factor associated with disease risk also received attention in this study. A relatively higher risk of hyperuricemia was observed among US citizens with higher education (college or above) in stratified analysis. Subgroup analysis showed that people with a college education or above and less than high school were associated with hyperuricemia. At present, the effects of education levels on hyperuricemia were disparate in different regions or different studies. In developing countries, an increased risk of hyperuricemia had generally been observed among people with low to moderate levels of education, whereas people with higher levels of education had comparatively better metabolic health (70, 71). This may be due to the latter being more concerned about their health. However, a meta-analysis of 9 studies based on populations from China, the US and Japan showed that higher education level was associated with hyperuricemia (72). Pan et al. speculated that the reason for this phenomenon may be that people with higher levels of education may consume more processed foods (72). Additionally, education level was not analyzed in the recent studies on DII and hyperuricemia. Hence, considering the differences in economy, culture, living habits and other aspects in different countries and even regions, further targeted studies should be carried out on the relationship between education level and hyperuricemia.

The relationship between uric acid or hyperuricemia and kidney damage or kidney disease had attracted the attention of scientists for decades. For stratified analysis based on ACR in this study, it was found that moderate ACR levels were significantly associated with hyperuricemia. Although the complex mechanism between them was not fully understood, it was generally believed that uric acid played an important role in the occurrence and development of kidney disease. Studies had shown that uric acid can activate immune responses and promote the development of pro-inflammatory and pro-fibrotic for renal structural cells (73). On the other hand, renal insufficiency or renal disease can further affect uric acid excretion. Moreover, recent studies had shown that hyperuricemia increases the risk of renal insufficiency in diabetic patients. Therefore, the association between ACR and hyperuricemia was worthy of further study in order to better guide the prevention, diagnosis and treatment of the disease.

Recently, a few studies had indicated an association between DII and hyperuricemia. The populations used by Kim et al. (74), Ye et al. (47) Wang et al. (48) are from South Korea, China and the US, respectively. This study was also based on NHANES, but compared with Wang et al. (48), this study included a broader population, and the subgroup analysis and stratification analysis were more comprehensive. The results from Wang et al. (48) were consistent with the general trend of the results of this study, further validating the accuracy of the conclusions of this study. Kim et al. indicated that the risk of hyperuricemia was positively correlated with the DII among females (74), but no significant results were found for males, while Ye et al. suggested that the lower the DII scores, the lower the risk of hyperuricemia in all sexes (47). Likewise, another study by Wang et al. found a positive association between DII and hyperuricemia in US adults (75), but the conclusion needed to be verified by more prospective studies, whereas the present study included more data and analyzed the relationship between DII and hyperuricemia at different levels, and concluded that there was a “U-shaped” relationship between DII and hyperuricemia. In contrast, none of the subgroup analyses in the analysis of Wang et al. were significant, compared with our subgroup analyses that found significance in the subgroups of age, gender, and hypertension. In addition, the results of Wang et al. showed a linear relationship between DII and hyperuricemia, while not discussing the analysis of it, which we doubt, and the robustness of the results should be more cautious. The conclusion that there was a “U-shaped” relationship between DII and hyperuricemia may be related to differences in the scope of inclusion and confounding factors.

### 4.1 Limitations

This study had several limitations. Firstly, the participants included in this study were over 20 years of age, and these outcomes may not be generalizable to the younger age group. Secondly, the observational nature of this study did not establish causality. Thirdly, because place of residence also had an impact on dietary structure, but the NHANES data did not provide information on the place of residence of the participants, we were unable to conduct analyses of the effect of place of residence on diet and hyperuricemia. Finally, dietary information was assessed once within 24h in the NHANES databases, the variability of daily diet was not accounted for, and measurement error could still be present in the self-reported diet. Therefore, more high-quality prospective studies were needed to verify the correlation between hyperuricemia and the DII.

## 5 Conclusion

In summary, this study showed that the risk of hyperuricemia increased at slightly higher DII scores (i.e., with pro-inflammatory diets), but not significantly at lower levels (i.e., with anti-inflammatory diets). Higher DII scores were significantly associated with a higher risk of hyperuricemia. Controlling the intake of pro-inflammatory foods may help reduce the risk of hyperuricemia, dietary modifications may be a potential way to prevent and control hyperuricemia. These factors, including sex, BMI, alcohol consumption, hypertension, diabetes, education level and ACR, may be one of the important risk factors leading to hyperuricemia. These results alert the public that pro-inflammatory diets may increase the risk of developing hyperuricemia, but further research is needed to confirm this conclusion. Nonetheless, this study provides some indications for the prevention of hyperuricemia and the burden of disease.

## Data availability statement

The original contributions presented in the study are included in the article/supplementary material. Further inquiries can be directed to the corresponding authors.

## Author contributions

XL: Data curation, Formal analysis, Investigation, Methodology, Project administration, Resources, Software, Validation, Writing – original draft, Writing – review & editing. T-YC: Data curation, Formal analysis, Methodology, Project administration, Resources, Writing – original draft. T-YG: Data curation, Formal analysis, Funding acquisition, Investigation, Project administration, Writing – original draft. K-QS: Data curation, Formal analysis, Methodology, Resources, Software, Writing – original draft. F-QY: Data curation, Formal analysis, Funding acquisition, Methodology, Project administration, Writing – original draft. Y-XY: Conceptualization, Data curation, Investigation, Methodology, Project administration, Resources, Software, Validation, Writing – original draft, Writing – review & editing. CZ: Conceptualization, Data curation, Methodology, Project administration, Resources, Software, Validation, Visualization, Writing – original draft, Writing – review & editing.
